# From the *Shenzhen Code* to the *Madrid Code*: New rules and recommendations for naming algae, fungi, and plants

**DOI:** 10.1002/ajb2.70026

**Published:** 2025-04-04

**Authors:** Nicholas J. Turland

**Affiliations:** ^1^ Botanic Garden and Botanical Museum Berlin Freie Universität Berlin Königin‐Luise‐Str. 6–8 Berlin 14195 Germany

**Keywords:** derogatory names, DNA sequences as types, institutional votes, International Botanical Congress, International Code of Nomenclature, *Madrid Code*, nomenclature, offensive names, proposals to amend the Code, registration of names and type designations

## Abstract

**Premise:**

A universally understood, precise, and stable system of naming organisms is essential for effective scientific communication. The *International Code of Nomenclature for algae, fungi, and plants*, of which the most recently published edition is the *Shenzhen Code* of 2018, provides this system for algae, fungi, and plants. This *Code* is regularly revised at an International Botanical Congress (IBC), usually held every 6 years, most recently in Madrid, Spain, in July 2024. The Madrid IBC amended the *Shenzhen Code*, and the changes took effect on 27 July 2024, when the closing plenary session of the IBC approved the decisions of the Nomenclature Section. It is important to promptly publicize this information because the new edition of the *Code* resulting from these amendments, the *Madrid Code*, will not be published until mid‐2025.

**Methods:**

I selected some of the more important of the 433 published proposals to amend the *Code* at the Madrid IBC. I sourced details from the proposals themselves, the “Synopsis of Proposals” and the “Report of Congress Action” (all published in the journal *Taxon*) and from the records made during the Nomenclature Section in Madrid.

**Results:**

For a general botanical audience, I discuss the background, outcomes (acceptance or rejection), and consequences of acceptance of the proposals.

**Conclusions:**

This commentary supplements the technical reports already published and provides an overview of some of the new or amended rules and recommendations in the upcoming *Madrid Code*.

The purpose of biological nomenclature, the naming of organisms, is to provide a means of reference to facilitate communication about those organisms. A universally understood, precise, and stable system of naming organisms is essential for effective scientific communication. The rules and recommendations of the international codes of nomenclature provide this system. One of these codes governs the nomenclature of algae, fungi, and plants, while other codes cover cultivated plants, animals, prokaryotes, prokaryotes described from sequence data, and viruses (see https://www.iaptglobal.org/other-codes). This commentary concerns the *International Code of Nomenclature for algae, fungi, and plants*, hereafter referred to simply as the *Code*.

The *Code* governs the nomenclature of all organisms traditionally treated as algae, fungi, or plants, whether fossil or non‐fossil, including blue‐green algae (*Cyanobacteria*), chytrids, oomycetes, slime molds, and photosynthetic protists with their taxonomically related non‐photosynthetic groups. The rules of the *Code* are not law but are voluntarily followed with an international consensus; i.e., the majority of botanists, mycologists, and phycologists worldwide have agreed to follow the rules. Scientific names published in compliance with these rules can achieve international acceptance. Breaking the rules might cause you some embarrassment, but there are no fines or worse punishments.

Anyone who has read the *Code* would agree that it is a very complex document. This complexity has evolved over 17 editions beginning with Alphonse de Candolle's *Lois de la Nomenclature Botanique* (Candolle, 1867). Throughout this more than 150‐year history, diverse issues have arisen prompting scientists to refine the rules at an International Botanical Congress (IBC), each time making the *Code* more relevant to current practice, more fit for purpose, and a little more precise but at the same time a little more complex.

The last edition of the *Code* to be published was the *Shenzhen Code* (Turland et al., 2018), which resulted from changes made by the 19th IBC held in Shenzhen, China, in July 2017. Seven years later, the *Code* was again amended by the 20th IBC held in Madrid, Spain, in July 2024. The Madrid IBC was unusual in that 7 years elapsed between it and the previous IBC in Shenzhen. Normally IBCs are held every 6 years. The 20th IBC was originally to be held in Rio de Janeiro, Brazil, in July 2023, but partly due to the Covid‐19 pandemic, it was postponed to 2024 and moved to Madrid.

Between July 2020 and October 2023, 167 authors published 433 proposals to amend the *Code* in *Taxon*, the journal of the International Association for Plant Taxonomy (IAPT). Each proposal was jointly reviewed and edited by John H. Wiersema and me, serving as Vice‐rapporteur and Rapporteur‐général, respectively, for nomenclature at the Madrid IBC. In February 2024, we published a “Synopsis of Proposals,” a compilation of all 433 proposals with the Rapporteurs' comments on each one (Turland and Wiersema, 2024).

Once the Synopsis of Proposals was public, all proposals were included in the mail vote, a preliminary guiding vote that advises the Section of the level of support for proposals. Those entitled to vote are (1) members of the IAPT, (2) authors of the proposals, and (3) members of the Permanent Nomenclature Committees (listed in Division III Provision 7.1 of the *Shenzhen Code*). Any proposal that receives 75% or more “no” votes in the mail vote is automatically rejected at the IBC unless at least six people want to reintroduce it for discussion.

The Nomenclature Section (“Section”) of an IBC takes place over 5 days in the week before the main part of the IBC. At this time, proposals to amend the *Code* are deliberated and voted on. In Madrid, the Section took place on 15–19 July 2024 in the conference hall of the central campus main building of the Consejo Superior de Investigaciones Científicas (CSIC). There were 173 registered members of the Section attending in person, although at any one time the number was lower, perhaps around 110–120 members (based on my rough counts during the Section and from photographs).

An anonymous survey of members of the Section revealed some interesting statistics on gender balance and taxonomic expertise. Printed questionnaires were distributed to all 173 members, of which 87 were completed and returned, representing a 50% response. Among the responding members, gender was 57% male, 37% female, and 6% using a different term or preferring not to answer. Taxonomic expertise was 78% in vascular plants, with no more than 8% of responding members declaring expertise in any one of algae, fungi, bryophytes, fossils, or “other.” Next time, I would like to include age and/or career stage in such a survey. In Madrid, Sandra Knapp (President of the Section) asked members in the auditorium to stand up if they were attending for the first time, and about half stood up, many of them apparently in their twenties or thirties. So it seems that a new generation of scientists, with a more even gender balance, is becoming involved in nomenclature, which is a very positive thing!

The entire Madrid Section was livestreamed on the internet. It was thus possible to observe the deliberations and voting but not to make comments or to vote. Altogether 219 unique users accessed the livestream at some point during the 5 days. Access times per user varied between 2 minutes and whole duration of the Section. There was no charge to register for or use this service, which was financially supported by the CSIC and the IAPT.

What of the fate of the 433 proposals to amend the *Code* in Madrid? Of the 134 proposals (31%) accepted, 26 were amended before they were accepted. Of the 272 (63%) rejected, 11 were rejected automatically because the proposal was dependent on another proposal that was rejected, and 106 were ruled as rejected because they received 75% or more “no” votes in the mail vote. Twenty‐two proposals were referred to the Editorial Committee of the *Madrid Code*, one proposal was referred to a special‐purpose committee (see below), and four proposals were withdrawn by their authors. In addition, 14 “floor proposals,” i.e., new proposals made at the Section and not previously published in *Taxon*, were dealt with during the final session on the morning of Friday, 19 July. Of these, seven were accepted, six were rejected, and one was withdrawn by its author.

Full details of the Section in Madrid, its decisions on proposals to amend the *Code*, its establishment of special‐purpose committees, and its appointments of the Rapporteur‐général and members of the Permanent Nomenclature Committees, are provided in the full, technical “Report of Congress Action” published in September 2024 in *Taxon* (Turland et al., 2024).

The *Madrid Code* is expected to be published in mid‐2025 in the IAPT's *Regnum Vegetabile* series, which is now published by the University of Chicago Press. Even though publication is still several months away, all but three of the new rules of the *Madrid Code* came into effect on 27 July 2024 when the decisions of the Section were approved at the closing plenary session of the IBC. The other three new rules have a starting date of 1 January 2026.

If the *Madrid Code* is already in effect, but not yet published, how can users know the new provisions? They can be found by using the “Report of Congress Action” (Turland et al., 2024) in conjunction with the “Synopsis of Proposals” (Turland and Wiersema, 2024), but this method is cumbersome. One of the main reasons for my writing the present commentary, therefore, was to offer a more user‐friendly overview. Here I will discuss a selection of proposals arranged into categories. The selection is somewhat eclectic, but I have tried to include some of the more important proposals discussed in Madrid. A fuller summary of changes to the *Code* is provided in the Preface of the *Madrid Code* itself (Turland et al., 2025).

## DNA SEQUENCES AS (OR INSTEAD OF) TYPES

Note that for a “name” to have any status under the *Code* it must be *validly published*, which is achieved by complying with various rules (listed in Article 6.3). A name that is not validly published (often called an invalid name, nomen invalidum, or nom. inval.) has no priority, no type, and cannot be the correct name for a taxon. Valid publication creates a name but does not imply any taxonomic circumscription beyond the inclusion of the type of the name. A *type* is a specimen or illustration that fixes the application of a name to a taxon. Under the current rules, a type can be a permanently preserved specimen (e.g., herbarium sheet, box, packet, jar, or microscope slide), a culture of an alga or fungus preserved in a metabolically inactive state (e.g., by lyophilization or deep freezing), or an illustration (in which case several restrictions apply). A type cannot be a DNA sequence, though, at least not yet.

Two sets of proposals resulted from the discussions of the Special‐purpose Committee on DNA Sequences as Types established at the Shenzhen IBC. The first set (Thiele et al., 2023a) would have provided for the future valid publication of names of microscopic algae or microfungi with DNA sequences as types; the second set (Thiele et al., 2023b) would have done the same for names of certain taxa other than vascular plants or bryophytes without nomenclatural types (so‐called typeless names) by fixing their application using DNA reference sequences. Both sets, each with 10 proposals, were rejected in the mail vote. Members of the Section did not reintroduce any of the proposals for discussion.

The rejection in the mail vote was probably largely due to the Nomenclature Committee for Algae (NCA) and the Nomenclature Committee for Fungi (NCF) not supporting the proposals, as was reported in the Synopsis of Proposals. These committees are influential, and proposals they support are often accepted at the Section, but those they do not support are usually doomed. Nevertheless, a new Special‐purpose Committee on Typeless Names and DNA Sequences as Types was established to further consider these issues and report back to the 21st IBC in Cape Town, South Africa, in 2029.

Meanwhile, mycologists were waiting to deliberate on a separate set of proposals specific to names of fungi at the 12th International Mycological Congress (IMC) in Maastricht, The Netherlands, in August 2024. Although the *Code* may be modified only at an IBC, Chapter F, which applies to names of organisms treated as fungi, may be amended only at an IMC. Two sets of proposals were considered at the Fungal Nomenclature Session of the 12th IMC on 15 August. One would have permitted DNA sequences to serve as types of fungal names, whereas the other would have permitted genomes to serve as types. The first set was withdrawn, and the second set was referred to yet another Special‐purpose Committee to report at the 13th IMC in Incheon, South Korea, in 2027. Hopefully, these two new Special‐purpose Committees will work together.

## REGISTRATION OF ALGAL AND PLANT NAMES (AND TYPE DESIGNATIONS)

It could be advantageous if new names of organisms were routinely added to open‐access, online databases with details of their authors, place and date of publication, and type, especially when names are published in less‐discoverable places, such as regional and/or print‐only journals and books. For some groups, nomenclatural indexing centers continuously monitor journals and books and record these details, as done, for example, by the International Plant Names Index (IPNI; https://www.ipni.org/) for names of vascular plants. *Registration* goes further than indexing by encouraging (or requiring) the authors of names and designations of types to deposit the names and associated data in a nomenclatural repository.

New names of fungi published since 2013 (including lichenized fungi and fossil fungi) are not validly published unless they are registered before publication in one of three nomenclatural repositories recognized by the *Code*: MycoBank (https://www.mycobank.org/), Index Fungorum (https://www.indexfungorum.org/), and Fungal Names (https://nmdc.cn/fungalnames/). Similarly, new designations of types (lectotypes, neotypes, and epitypes) published since 2019 for names of fungi are not effective unless they are registered. When a name or type designation is registered, the repository issues an identifier (e.g., 564515), which must be cited together with the name or type designation when it is first published. This is *mandatory* and *proactive* registration.

The Registration Committee, one of the new Permanent Nomenclature Committees created at the Shenzhen IBC of 2017, discussed proposals to extend this mandatory and proactive registration to the names of algae and plants, so that it would apply to all organisms covered by the *Code*. However, the Committee voted against making such proposals, as explained in its report (Loizeau et al., 2023). Instead, two members of the Committee prepared a set of proposals (Brinda and Watson, 2023) to augment Article 42 to provide a clear mechanism for *voluntary* and *retrospective* registration of algal and plant names and type designations, i.e., for registration to be done after valid publication of a name or after publication of a type designation. The proposals were accepted, resulting in several new provisions in Article 42 and a new Recommendation 42A.

A subsequent floor proposal expanded the new provisions in Article 42 to allow for other mechanisms of registration of new names, e.g., proactive registration. However, regardless of the mechanism used, registration for names of algae and plants will remain voluntary and not be a condition of effective and/or valid publication.

## VIRTUAL PARTICIPATION IN THE NOMENCLATURE SECTION

The Special‐purpose Committee on Virtual Participation in the Nomenclature Section, established at the Shenzhen IBC, submitted nine proposals to amend Division III (Landrum et al., 2021). All except one of these proposals was rejected. The successful proposal resulted in a new Recommendation 2 (in Provision 4), as follows:

“Individuals or groups should be able to observe the Nomenclature Section of an International Botanical Congress online on the World Wide Web. The Organizing Committee of the International Botanical Congress in consultation with the Bureau of Nomenclature should be responsible for ensuring that this is implemented.”

In practice, Recommendation 2 would advise, but not require, that the Section be livestreamed so that people can at least observe it. The Section in Madrid was livestreamed exactly in this way. If the Organizing Committee of the IBC had the primary responsibility for making such a livestream happen, the most important challenge would be securing the necessary financial support, which in Madrid was generously provided by the CSIC and the IAPT.

## INSTITUTIONAL VOTES

The Committee on Institutional Votes is another Permanent Nomenclature Committee created at the Shenzhen IBC. Its role is to maintain a list of taxonomically active institutions with herbaria, e.g., universities, botanical gardens, research centers, and give them the right to vote on proposals at an IBC by allocating them institutional votes. Any member of the Section can be authorized to carry up to 14 institutional votes from up to 14 institutions. That member need not be affiliated with any of these institutions but must have explicit authorization from each institution to carry and use the votes. Institutions are thus able to have a say in important decisions made by the Section, even if they cannot send any of their own staff to the IBC, which could well be the case for less wealthy institutions.

While updating the list before the Madrid IBC, the Committee was concerned that there was no established method for correlating an institution's level of taxonomic activity with its number of votes, which could range from one to a maximum of seven. It would have been necessary to measure the activity of each of the more than 500 institutions allocated votes, establish seven bands of taxonomic activity, and reallocate votes on that basis. The Committee was also aware that larger institutions with more votes could enjoy a double advantage if they had the financial resources to send more members of staff to an IBC, where each member of the Section has one personal vote independent of any institutional votes. Analysis of the geographic distribution of institutional votes showed that the larger institutions were mostly in the Global North, especially in Europe, whereas Africa had the lowest number (Ulloa Ulloa et al., 2024). The consistent allocation of institutional votes as well as the geographical imbalance could be improved by allocating only one institutional vote to each institution regardless of its size. This new principle, one institution, one vote, was proposed by the Committee (Ulloa Ulloa et al., 2023) and was accepted in Madrid.

At future IBCs, the 572 institutions that were on the list for the Madrid IBC (Ulloa Ulloa et al., 2024) will each be allocated one vote, unless they are no longer active or have been merged into other institutions. An institution not already on the list but wishing to be added should “notify the Rapporteur‐général of its wish to be allocated a vote and provide relevant information regarding its current level of taxonomic activity (e.g., active staff, collections, publications) and show that it is registered in an online, open‐access international or regional index of herbaria, collections, or institutions” (Division III Provision 3.2, as amended in Madrid).

The index in which an institution needs to be registered could be, e.g., Index Herbariorum (https://sweetgum.nybg.org/science/ih/). Essentially, any institution registered in an index as specified and that is taxonomically active will be allocated one vote, but it is essential to apply for that vote well in advance of the IBC. The Committee on Institutional Votes will revise the list, but it must also be approved by the General Committee before the IBC is held (Division III Provision 3.1). The approval takes at least several weeks, meaning that last‐minute applications cannot be considered. However, a staff member of an institution can apply in person at the Section for a vote for their own institution (Division III Provision 3.4).

## OFFENSIVE NAMES AND OTHER CULTURALLY SENSITIVE ISSUES

### Epithets such as “*caffra*” are changed to *afra*

Perhaps the most talked‐about change to the *Code* in Madrid was the acceptance of the proposals by Smith and Figueiredo (2021) to permanently and retroactively eliminate the racial offense inherent in epithets such as *caffra*, which refer to a region of southern Africa and its inhabitants and derive from a noun that is a racial slur in languages such as Afrikaans and English. Use of this racial slur, which is comparable to the “N‐word” in English, is legally actionable in South Africa. The proposal accepted in Madrid changed the spelling of epithets with the root *caf[e]r‐* or *caff[e]r‐* so that the initial “*c*” is dropped and “*ff*,” if present, becomes “*f*.” Epithets so changed include *cafer*/*caffer* → *afer*; *cafferiana* → *aferiana*; *caffra*/*cafra* → *afra*; *caffrae* → *afrae*; *caffraria*/*cafraria* → *afraria*; *caffrorum* → *afrorum*; *caffrum* → *afrum*. The original spellings with a “*c*” are treated as orthographical variants, so that there is no change in author, place and date of publication (and hence priority), or type. The names are not rejected, so their continued use is assured albeit with a spelling based on the root *af[e]r‐*. An actual example is the name *Erythrina caffra* Thunb. (Fabaceae), the coast coral tree or African coral tree, which is now *E. afra* Thunb.

The proposal was accepted by an anonymous ballot in which members wrote “yes” or “no” on voting cards and deposited them in one of the ballot boxes provided. The result was 351 yes, 205 no (=63% yes, acceptance requiring ≥60% majority).

The acceptance of this proposal in Madrid on 18 July was reported the same day in an article in *Nature News* (Callaway, 2024), in which it was incorrectly stated that “the coast coral tree will, from 2026, be formally called *Erythrina affra*, instead of *Erythrina caffra*.” First, the new rule became effective upon acceptance of this resolution to approve the decisions of the Section at the closing plenary session of the IBC on 27 July 2024, not “from 2026” because no later starting date is specified in the new rule. Second, according to the new rule, the epithet is to be spelled *afra*, i.e., with a single letter “*f*,” not “*affra*.”

Smith and Figueiredo (2021, see Supporting Information) provided an Excel file listing 218 validly published names of vascular plants and bryophytes that have the root *caf[e]r‐* or *caff[e]r‐*. These authors also stated that they had found 13 names of algae and 70 names of fungi similarly affected, making a total of 301 names of algae, fungi, and plants. Of the 218 names of vascular plants and bryophytes, the authors stated that 56 were accepted and in current use, 155 were synonyms, and seven were unplaced.

I have scanty information about the number of names of fossil taxa affected, but a search of the International Fossil Plant Names Index (https://ifpni.org/) found *Mohria* “*caffrorites*” L. G. Markova et al. (a fossil fern spore), the epithet of which derives from that of *Mohria* “*caffrorum*” (L.) Desv. These names are now spelled *M. afrorites* and *M. afrorum*, respectively [the latter is now *Anemia afrorum* (L.) Christenh., Schizaeaceae].

It is important to emphasize that the new rule in Article 61 applies only to names with these particular epithets. Other names are not at risk of being changed in a similar way. Eliminating other epithets would require another proposal to amend the *Code* at a future IBC, first surviving the mail vote and then being accepted by the Section.

### Mechanism to reject derogatory names

The Section discussed a set of four proposals by Hammer and Thiele (2021), which aimed to create a mechanism to allow the rejection of culturally offensive and inappropriate names. Rejection is a formal process (Article 56) that adds a name to the list of suppressed names that are not to be used (Appendix V). Of the four proposals, two were accepted with amendments, as follows:

*Add a new Article 51.2:*

*51.2*. Despite Art. 51.1, a legitimate name of a new taxon or a replacement name published on or after 1 January 2026 may be rejected (under Art. 56.1) because it, or its epithet, is derogatory to a group of people.

*Amend the first sentence of Article 56.1 as follows (new text in bold):*

*56.1*. Any name that would cause a disadvantageous nomenclatural change (Art. 14.1) **or that is derogatory to a group of people (Art. 51.2)** may be proposed for rejection.

Hence, under the new Article 51.2, a name must comply with the following conditions to be proposed for rejection:

- published on or after 1 January 2026 (names published before 2026 are not eligible).
- legitimate (illegitimate names are not eligible).
- name of a new taxon or a replacement name (new combinations and names at new rank, i.e., names that have a basionym, are not eligible).
- derogatory to a group of people.

The 2026 starting date means that only names published from 2026 onward can be proposed for rejection. It does not mean that from 2026 onward, names can be proposed for rejection regardless of when they were published. Names not validly published (“invalid” names) are also not eligible because they can never be legitimate. The Oxford English Dictionary (https://www.oed.com/) defines derogatory as “having the effect of lowering in honour or estimation; depreciatory, disparaging, disrespectful, lowering.”

Any future proposals to reject derogatory names will go through the same process as any other rejection proposal. This process consists of the following stages: the proposal is published in *Taxon*; the specialist committee for the group concerned (e.g., the Nomenclature Committee for Vascular Plants) considers the proposal and recommends for or against rejection; the General Committee considers the proposal, approves or overturns the specialist committee's recommendation, and makes its own recommendation; at this point, the name becomes rejected and is added to Appendix V subject to approval by a vote at a later IBC.

### Recommendation not to publish offensive or inappropriate names

Not only would the prospect of rejection loom over a new, derogatory name, but a proposal accepted in Madrid (Mosyakin, 2021) added an explicit Recommendation to the *Code* warning authors to avoid such names (Recommendation 51A): “Authors are strongly advised to avoid publishing names of new taxa or replacement names that could be viewed as inappropriate, disagreeable, offensive, or unacceptable by any national, ethnic, cultural, or other groups.”

### Special‐purpose Committee on Ethics in Nomenclature

The Section in Madrid also established a “Special‐purpose Committee on Ethics in Nomenclature” with the mandate to consider ethical issues associated with naming taxa and to report back to the Cape Town IBC in 2029. This mandate, together with the membership of the Committee, is still to be finalized by the General Committee. A proposal to insert a new Chapter E consisting of a code of ethics (Malavasi and Škaloud, 2022) was referred to this Special‐purpose Committee.

## AUTHOR CONTRIBUTIONS

N.J.T. was responsible for the conceptualization and methodology, wrote the original manuscript, and revised the manuscript as recommended by the reviewers.
